# Weighted gene co-expression identification of CDKN1A as a hub inflammation gene following cardiopulmonary bypass in children with congenital heart disease

**DOI:** 10.3389/fsurg.2022.963850

**Published:** 2022-08-24

**Authors:** Huan Chen, Jinglan Liu, Yuqing Wu, Li Jiang, Mi Tang, Xin Wang, Xiaoling Fang, Xi Wang

**Affiliations:** ^1^ Department of Obstetrics and Gynecology, The Second XIANGYA Hospital Of Central South University, Changsha, China; ^2^ Department of Obstetrics and Gynecology, Zhu Zhou Hospital Affiliated to Xiangya school of medicine, CSU, Zhuzhou, China; ^3^ Department of cardiovascular surgery, The Second XIANGYA Hospital Of Central South University, Changsha, China

**Keywords:** cardiopulmonary bypass, WGCNA, hub genes, CDKN1A, congenital heart disease

## Abstract

**Background:**

Congenital heart disease (CHD) is the most common type of birth defect. Most patients with CHD require surgery, and cardiopulmonary bypass (CPB) is the most common surgery performed.

**Methods:**

The present study utilized weighted gene co-expression network analysis (WGCNA) to identify key inflammation genes after CPB for CHD. The GSE132176 dataset was downloaded from the Gene Expression Omnibus(GEO) database for WGCNA to identify the modules closely related to clinical traits. Disease enrichment, functional annotation and pathway enrichment were performed on genes in the module closely related to clinical traits using Enrichr and Metascape. Immune infiltration analysis was also performed on the training dataset using CIBERSORT. Finally, we identified hub genes using high gene significance (GS), high module members (MMs) and Cytoscape, and we verified the hub genes using an independent dataset and Western blot analysis.

**Results:**

WGCNA showed that the brown module with 461 genes had the highest correlation to CHD after CPB. Functional annotation and pathway enrichment analysis were performed using Gene Ontology (GO) and Kyoto Encyclopedia of Genes and Genomes (KEGG) analyses, which showed that genes in the brown module were enriched in inflammation-related pathways. In the disease enrichment analysis, genes in the brown module were enriched for inflammatory diseases. After the 30 most highly associated brown intramodular genes were screened, a protein-protein interaction network was constructed using the STRING online analysis website. The protein-protein interaction results were then calculated using 12 algorithms in the cytoHubba plugin of Cytoscape software. The final result showed that *CDKN1A* was the fundamental gene of post-CPB for CHD. Using another independent validation dataset (GSE12486), we confirmed that *CDKN1A* was significantly differentially expressed between preoperative and postoperative CPB (Wilcoxon, *P* = 0.0079; T-test, *P* = 0.006). In addition, *CDKN1A* expression was elevated in eosinophils, neutrophils, memory CD4 T cells and activated mast cells. Western blot analysis showed that the expression of CDKN1A protein was significantly higher postoperative CPB than preoperative CPB. Moreover, *CDKN1A* was mainly related to inflammation.

**Conclusion:**

In summary, we found a relationship between *CDKN1A* and inflammation after CPB for congenital heart disease by WGCNA, experiments and various bioinformatics methods. Thus, *CDKN1A* maybe serve as a biomarker or therapeutic target for accurate diagnosis and treatment of inflammation after CPB in the future.

## Introduction

Congenital heart disease (CHD) refers to cardiovascular malformations resulting from abnormal fetal cardiovascular development (1). CHD is the most common congenital defect, affecting 0.5%–0.8% of all live births (2). Approximately 25% of infants with CHD require invasive treatment in the first year of life (3). Currently, most cardiac surgeries are performed with the support of cardiopulmonary bypass (CPB) (4), which is a technique that takes over cardiopulmonary function during surgery to maintain blood circulation and oxygen supply (5). CPB is required during surgery for CHD, especially cyanotic CHD (6). The increasing employment of CPB technology has greatly improved cardiovascular surgery and significantly increased the survival rates of patients (7). However, there is a significant contribution to postoperative morbidity and mortality from cardiopulmonary bypasses (CPB), especially in pediatric cardiac surgery (8). The use of CPB during cardiac surgery significantly changes the internal environment due to anesthesia, CPB, surgical operations and other factors (9). CPB during cardiac surgery causes a systemic inflammatory response with cytokine secretion (10) similar to local reactions after tissue damage (11). The most frequent complications of cardiovascular surgery are associated with CPB and its inflammatory response (12), e.g., respiratory complications (13) and postoperative atrial fibrillation (POAF) (14). CPB can also lead to systemic inflammatory response syndrome (SIRS), a condition that is clearly reviewed in congenital cardiac surgery programs (15). Postoperative SIRS after CPB is triggered by the contact of blood with non-physiological artificial tubes, surgical injuries, anesthesia, body temperature changes, ischemia and reperfusion of organs (11, 16, 17). The use of oxygenators plays a major role in the postoperative effects of CPB (18). SIRS further causes multiple organ dysfunction syndrome (MODS) (19) and is the leading cause of morbidity and mortality in critically ill patients (20).

Gene expression analysis is the basis for identifying differentially expressed genes (DEGs) under two conditions (21) and also a method to explore the underlying molecular causes of these differences (22). Many strategies exist to analyze high-throughput omics data to explore the complexity that distinguishes the DEGs in biological groups. The most common analysis is based on differential expression, which is determined by the changes in molecular characteristics, either expression or abundance, between groups (23). Weighted gene co-expression network analysis (WGCNA), which was proposed by Langfelder and Horvath in 2008 (24), is a comprehensive and novel R software package, and it is widely used in genomics and bioinformatics research to obtain correlation patterns between genes and detect biomarkers or pathways (25). Compared to traditional differential gene expression analysis, WGCNA does not manage genes as a single entity but combines the interrelation of genes to produce a structure called a module (26).

**Figure 1:**
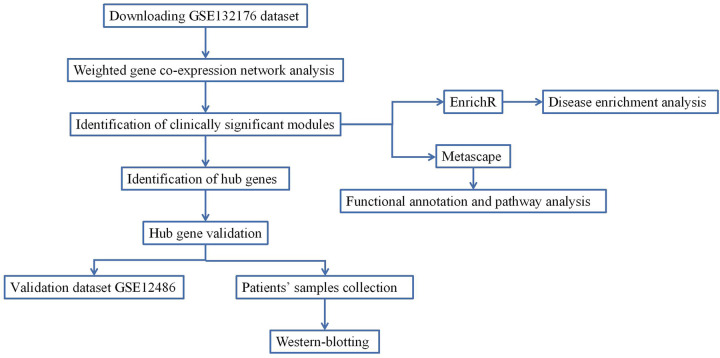
The workflow of data preparation, processing, analysis and validation.

In the present study, a dataset from the Gene Expression Omnibus (GEO) database was used to perform WGCNA to investigate the hub genes related to CPB for CHD. Figure 1 shows the study workflow, including data preparation, processing, analysis and validation.

## Materials and methods

### Data collection

The GSE132176 dataset (27) is based on the GPL13158 Affymetrix HT HG-U133+ PM Array Plate platform [HT_HG-U133_Plus_PM] and includes 40 biopsy specimens of right atrial pre-CPB (CPB_before) and post-CPB (CPB_after) from 10 patients with tetralogy of Fallot (TOF) and 10 patients with atrial septal defect (ASD). The GSE132176 dataset was used as a training set to construct co-expression networks to screen for modules closely related to post-CPB. The GSE12486 dataset (28) is based on the GPL570 Affymetrix Human Genome U133 Plus 2.0 Array [HG-U133_Plus_2] and includes 10 samples of left ventricular specimens before and after CPB, and it was used as a validation dataset. Both datasets were downloaded from the GEO database (http://www.ncbi.nlm.nih.gov/geo/).

### Data preprocessing

After downloading the normalized data of the GSE132176 dataset (containing a total of 54,716 genes) from the GEO database, a sample clustering method based on the Pearson correlation matrix was performed, and the average distance between different samples was used to evaluate the microarray quality. After discarding the outlier samples, the genes in the top 10% of the variance were screened from this dataset using R software.

### Co-expression network construction

We used the WGCNA package in R (25, 29) to construct a co-expression network based on the top 10% of the variance genes. First, the absolute value of the correlation coefficient between the two genes was utilized to construct the Pearson correlation matrix. The power function (amn = |cmn|*β*) was then used to construct a weighted adjacency matrix (cmn = Pearson correlation between gene m and gene n; mn = neighborship between gene m and gene n where *β* is a soft threshold parameter that emphasizes a strong correlation between genes). Finally, the most appropriate *β* value was selected to convert the adjacency matrix into a topological overlap matrix (TOM). The minimum size of the gene dendrogram was 30, and similar genes were split into one module.

### Identification of clinically significant modules

We next determined the modules related to clinical features. Depending on the linear regression between clinical traits and gene expression, the log10 conversion of the P value was defined as gene significance (GS), and the module significance (MS) was then calculated using the average value of all genes in one module. The module with the largest absolute value of MS was considered as the module with the closest relationship to clinical characteristics. Module eigengene (ME) is defined as the principal component of a given module, and it represents the gene expression profile in a module. Pearson correlations between modules and clinical features were then calculated, and modules highly relevant to a given clinical feature were selected for further analysis.

### Identification of hub genes

Hub genes were designated by two methods. First, genes with high GS and high module members (MMs) were defined as hub genes. The cutoff value was the GS absolute value >0.2 and the MM absolute value >0.95. Second, the softConnectivity function in WGCNA was used to calculate the top 30 genes with the highest connectivity in the module most closely related to clinical features. The results were then imported into the STRING online site (30) for protein-protein interaction (PPI) network analysis with a medium confidence score >0.4 as the cutoff criteria. Finally, the PPI network results were imported into Cytoscape (31) to calculate the top 10 genes for each algorithm using 12 algorithms in the cytoHubba plugin. The results of the two methods were overlapped to obtain the key genes.

### Functional annotation and pathway analysis

Functional annotation and pathway analysis of genes in modules most relevant to clinical traits were conducted using Metascape (https://metascape.org/gp/index.html), an online analysis site for gene and function enrichment (32). Enriched terms with a false discovery rate (FDR) <0.05 were considered to be significantly enriched terms.

### Disease enrichment

The Enrichr database (http://amp.pharm.mssm.edu/Enrichr/) is a comprehensive online tool for gene enrichment analysis that can be used for analysis and to download data. The Enrichr database contains a large library of genome annotations, such as Transcription, Pathways, Ontologies, Diseases/Drugs and Cell types. We used the Enrichr database for disease enrichment analysis.

### Immune infiltration analysis

The immune cell number of the GSE132176 dataset was calculated using the CIBERSORT package in R for assessing the differences in immune cells between the pre-CPB and post-CPB groups. The relationship of the hub gene with immune and inflammatory cells was also analyzed using the CIBERSORTx online analysis website (CIBERSORTx.stanford.edu).

### Hub gene validation

The hub gene expression was verified using the GSE12486 validation dataset. The expression matrix of key genes in the validation set was extracted, and the Wilcoxon & T.test was then used to compare the expression differences between the two groups.

### Patients and samples

From 1 January 2022 to 1 February 2022, two patients with TOF (1 male and 1 female with a mean age of 1 year) and two patients (1 male and 1 female with a mean age of 4.5 years) with ASD underwent surgical treatment at the Department of Cardiothoracic Surgery, Second Xiangya Hospital, Central South University. Right atrium specimens were collected from the patients before and after CPB for western blot analysis. The specimens were placed in liquid nitrogen immediately after collection and then transferred to −80°C for storage.

The study was approved by the Research and Clinical Trial Ethics Committee of Second Xiangya Hospital, and all eligible participants provided written informed consent. All procedures were performed in compliance with the ethical standards of the Declaration of Helsinki guidelines and relevant Chinese policies.

### Western blot analysis

Total proteins from heart tissue were extracted with radioimmunoprecipitation assay (RIPA) lysis buffer and separated by 12% sodium dodecyl sulfate-polyacrylamide gel electrophoresis (SDS-PAGE) at 70 V for 30 min and 110 V for 50 min using a blotting system (Bio-Rad, Hercules, CA). Proteins were then transferred to polyvinylidene difluoride (PVDF) membranes (0.45 µm), and the membranes were subsequently blocked with 5% skim milk in phosphate-buffered saline with Tween 20 (PBST) for 4 h. After blocking, the membranes were incubated with an anti-p21 antibody (1:500, Abcam ab227443) overnight at 4°C. The membranes were then washed and incubated with horseradish peroxidase-conjugated secondary antibody. β-actin levels (Abcam ab179467) were used as a loading control. The target bands were analyzed for grey-scale values by ImageJ software.

## Results

### Construction of weighted co-expression network and identification of key modules

**Figure 2:**
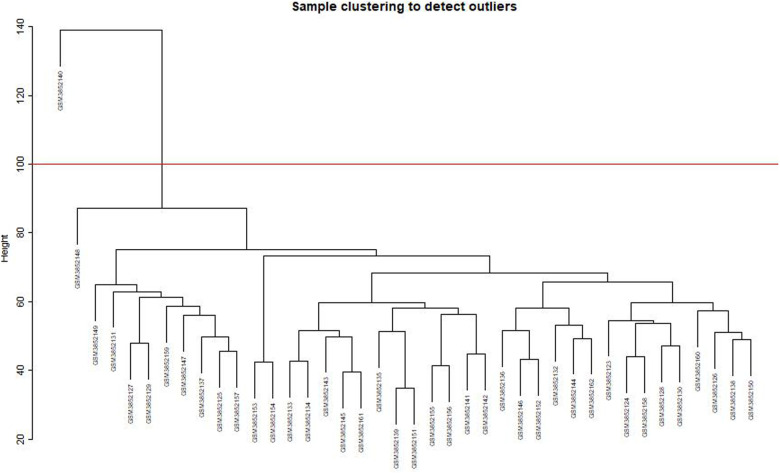
Sample clustering to detect outliers. GSM3852140 is considered a significant outlier and the data from this sample is excluded under the condition that abline equals 100.

**Figure 3:**
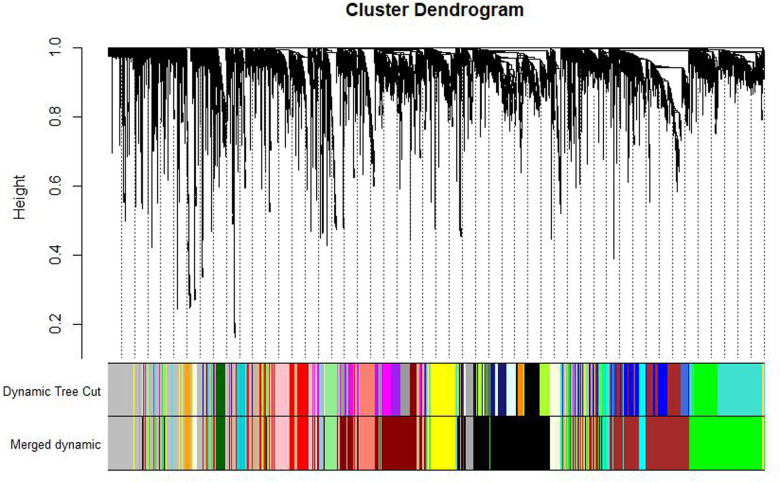
Gene dendrogram and module colors. After calculating eigengene, hierarchical clustering of modules, and merging the more similar modules, only 19 modules remain.

**Figure 4:**
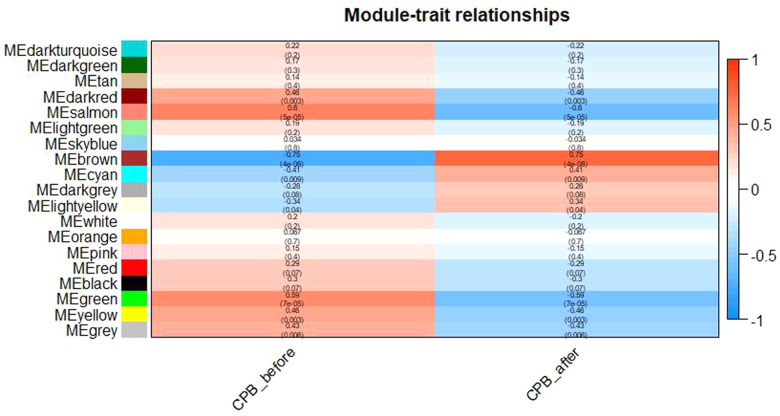
Diagram of Module-trait relationships. As shown in the figure, the brown module has the highest correlation with CPB_after(*P* = 0.75 × 10^4e−8^).

After clustering the samples, GSM3852140 was considered as a significant outlier, resulting in the inclusion of 39 samples with clinical data in the present study (Figure 2). The WGCNA package in R was then used to divide genes with similar expression patterns into one module. To ensure the normal operation of the scale-free network, we selected a suitable soft threshold value of 12 (scale-free *R*^2^ = 0.87). In total, 19 modules were identified (Figure 3), and the brown module had the highest correlation to CPB_after (*P* = 0.75 × 10^4e−8^) (Figure 4).

### Identification of hub genes

**Figure 5:**
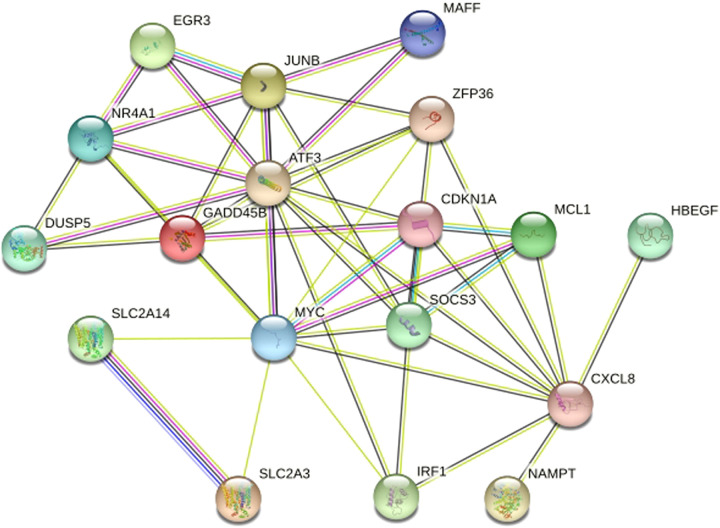
Protein protein Interaction Network Diagram. After removed disconnected nodes in the network, only 18 genes remained.They were *MCL1*, *JUNB*, *ZFP36*, *CDKN1A*, *NR4A1*, *MYC*, *IRF1*, *ATF3*, *MAFF*, *EGR3*, *SOCS3*, *GADD45B*, *DUSP5*, *CXCL8*, *SLC2A14,SLC2A3*, *NAMPT* and *HBEGF*.

**Figure 6:**
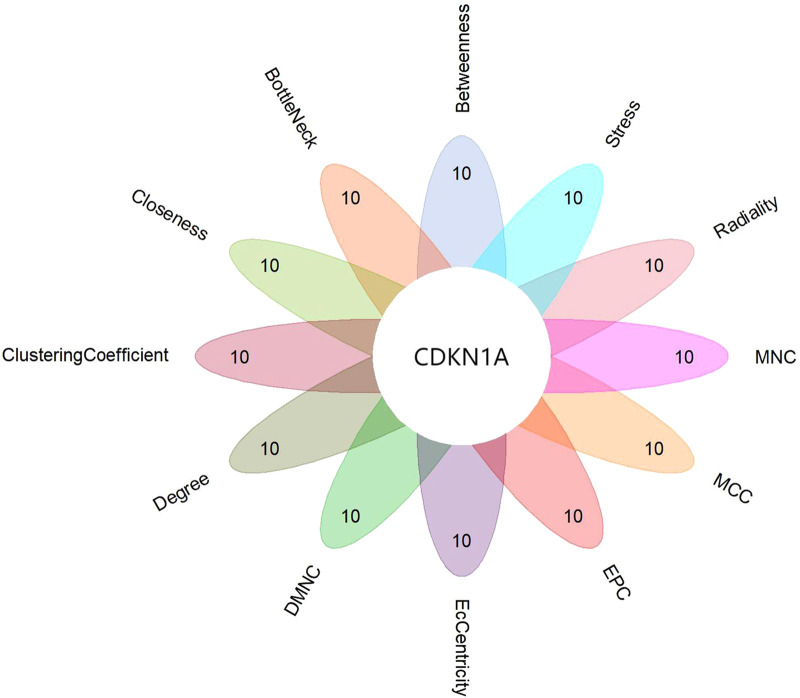
Flower plot for cytohubb results. Each petal represents one of cytohubb's algorithms, and only CDKN1A generemains after taking the intersection.

WGCNA selected 461 genes as brown modular genes. A total of six genes, namely, *CCNL1*, *CDKN1A*, *MAFF*, *SLC2A14///SLC2A3*, *SERTAD1* and *TNFRSF10D*, were screened in the brown module at a cutoff of GS absolute value >0.2 and MM absolute value >0.95. The 30 most highly connected genes in the brown module were *CCNL1*, *DNAJB1*, *MCL1*, *JUNB*, *ZFP36*, *CDKN1A*, *NR4A1*, *MYC*, *SLC2A3*, *IRF1*, *ATF3*, *IL8*, *MAFF*, *EGR3*, *SOCS3*, *GADD45B*, *DUSP5*, *CXCL8, SLC2A14///SLC2A3*, *SERTAD1*, *SLC25A25*, *TNFRSF10D*, *SOCS3*, *NAMPT* and *HBEGF* (only 26 remained after removing duplicates and no probe annotations). These 26 genes were then imported into the STRING online site for PPI network analysis (Figure 5), and the results were downloaded for further analysis. Using the 12 algorithms in the cytoHubba plugin of Cytoscape, the top 10 genes of each algorithm were calculated, and the results are shown in Table 1. Only *CDKN1A* was present in all 12 algorithms (Figure 6), and this gene was also present in the results of the former method.

**Table 1:** The top ten genes of each algorithm.

Rank	Betweenness	BottleNeck	Closeness	ClusteringCoefficient	Degree	DMNC	EcCentricity	EPC	MCC	MNC	Radiality	Stress
1	MYC	MYC	ATF3	MCL1	ATF3	ZFP36	ATF3	ATF3	MYC	ATF3	ATF3	MYC
2	ATF3	ATF3	MYC	IRF1	MYC	MCL1	IRF1	MYC	ATF3	MYC	MYC	ATF3
3	CXCL8	CXCL8	CXCL8	EGR3	CXCL8	IRF1	CXCL8	CXCL8	SOCS3	JUNB	CXCL8	CXCL8
4	JUNB	NR4A1	SOCS3	DUSP5	JUNB	CXCL8	SOCS3	SOCS3	CXCL8	SOCS3	SOCS3	JUNB
5	SOCS3	CDKN1A	JUNB	ZFP36	SOCS3	SOCS3	ZFP36	JUNB	JUNB	GADD45B	JUNB	SOCS3
6	GADD45B	MCL1	GADD45B	SLC2A3	GADD45B	CDKN1A	CDKN1A	GADD45B	ZFP36	CXCL8	ZFP36	NR4A1
7	NR4A1	NAMPT	ZFP36	CDKN1A	ZFP36	MYC	MYC	ZFP36	GADD45B	ZFP36	CDKN1A	GADD45B
8	CDKN1A	GADD45B	CDKN1A	SLC2A14	CDKN1A	GADD45B	MCL1	CDKN1A	CDKN1A	CDKN1A	GADD45B	CDKN1A
9	ZFP36	HBEGF	NR4A1	MAFF	NR4A1	NR4A1	NAMPT	NR4A1	NR4A1	NR4A1	IRF1	ZFP36
10	MCL1	IRF1	IRF1	NR4A1	MCL1	EGR3	GADD45B	IRF1	MCL1	MCL1	NR4A1	MCL1

### Gene Ontology (GO) and Kyoto Encyclopedia of Genes and Genomes (KEGG) pathway enrichment analyses

**Figure 7:**
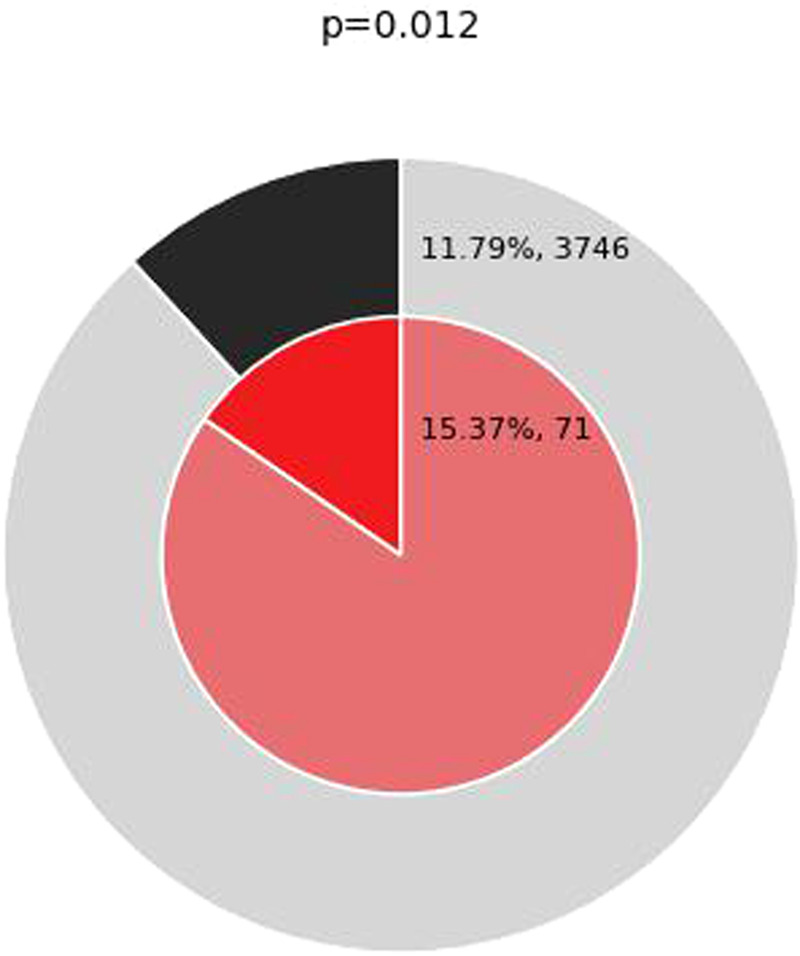
Enrichment of genes matching membership term: inflammation. The outer pie shows the number and the percentage of genes in the background that are associated with the inflammation (in black); the inner pie shows the number and the percentage of genes in the brown module gene list that are associated with the inflammation. The *P*-value indicates whether the membership is statistically significantly enriched in the brown module gene list.

**Figure 8:**
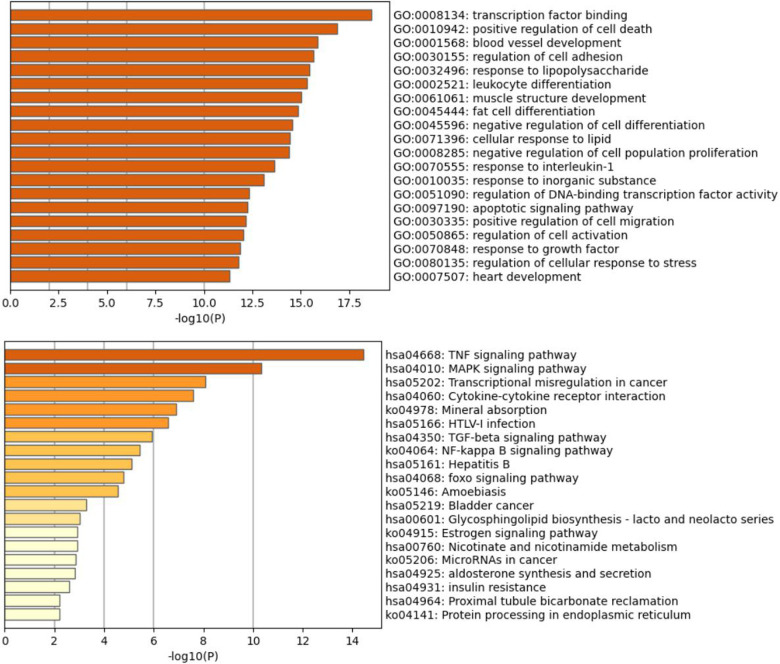
Bar graph of the top 20 GO and KEGG pathways (http://www.genome.jp/kegg/) across input brown module genes, colored by p-values.

GO and KEGG enrichment analyses were performed on the genes within the brown module, and we discovered that genes within the brown module were significantly enriched in GO terms and pathways associated with inflammation (Figure 7). The top 20 GO terms and the top 20 KEGG pathways (http://www.genome.jp/kegg/) are shown in Figure 8. The top 20 GO terms and the top 20 KEGG pathways that *CDKN1A* was involved in are shown in Table 2.

**Table 2:** The top 20 gene ontology and the top 20 KEGG pathway that CDKN1A was involved.

GO term	Description	KEGG term	Description
GO:0031960	response to corticosteroid	hsa05202	Transcriptional misregulation in cancer
GO:0010942	positive regulation of cell death	hsa05166	HTLV-I infection
GO:0071396	cellular response to lipid	hsa05161	Hepatitis B
GO:0048659	smooth muscle cell proliferation	hsa04068	Foxo signaling pathway
GO:0019900,	kinase binding	hsa05219	Bladder cancer
GO:0036294	cellular response to decreased oxygen levels	ko04915	Estrogen signaling pathway
GO:0046916	cellular transition metal ion homeostasis	ko05206	MicroRNAs in cancer

### Disease enrichment

**Figure 9:**
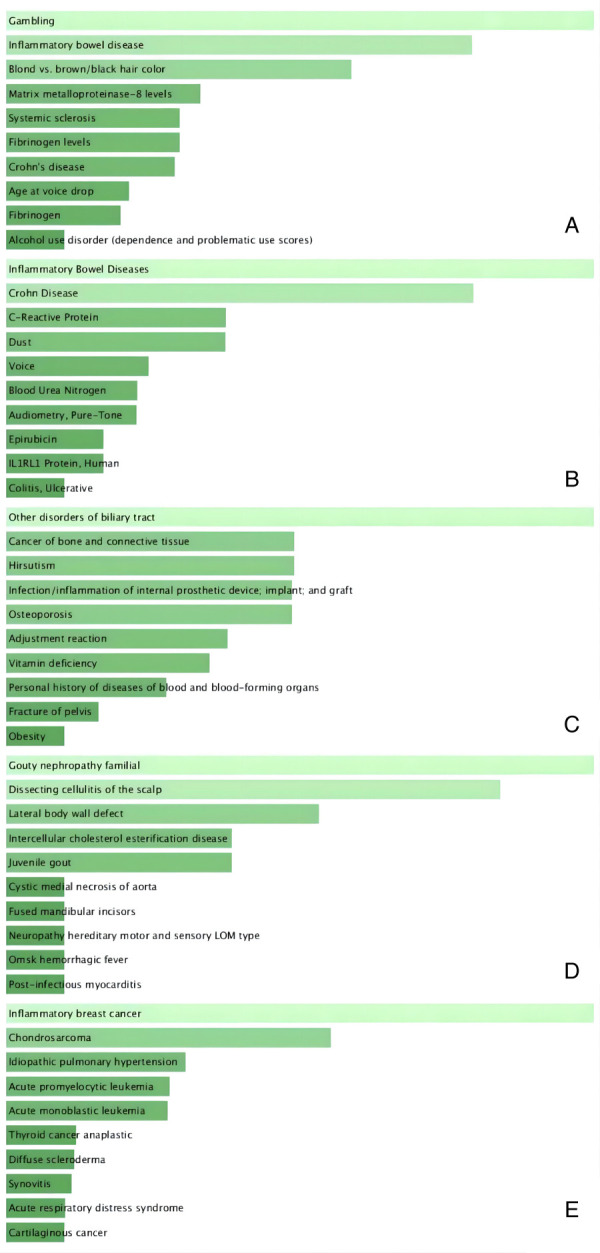
Disease enrichment plot. (**A**) GWAS_Catalog_2019, (**B**) PhenGenI_Association_2021, (C) PheWeb_2019, (**D**) Rare_Diseases_AutoRIF_ARCHS4_Predictions, (**E**) Rare_Diseases_GeneRIF_Gene_Lists. Ranked by *P*-values. The genes in the brown module that were significantly enriched in inflammatory diseases, including inflammatory bowel disease (**A,B**); infection/inflammation of internal prosthetic device, implant and graft (**C**); post-infectous myocarditis (**D**); inflammatory breast cancer (**E**).

We used the Enrichr online enrichment site to identify the genes in the brown module that were significantly enriched in inflammatory diseases (Figure 9). For example, inflammatory bowel disease in GWAS_Catalog_2019 (*P* = 1.01 × 10^−5^) and PhenGenI_Association_2021 (*P* = 0.0013), infection/inflammation of internal prosthetic device, implant and graft in PheWeb_2019 (*P* = 0.0036), post-infectous myocarditis in Rare_Diseases_AutoRIF_ARCHS4_Predictions (*P* = 5.89 × 10^−9^), inflammatory breast cancer in Rare_Diseases_GeneRIF_Gene_Lists (*P* = 5.98 × 10^−16^).

### Immune infiltration analysis

**Figure 10:**
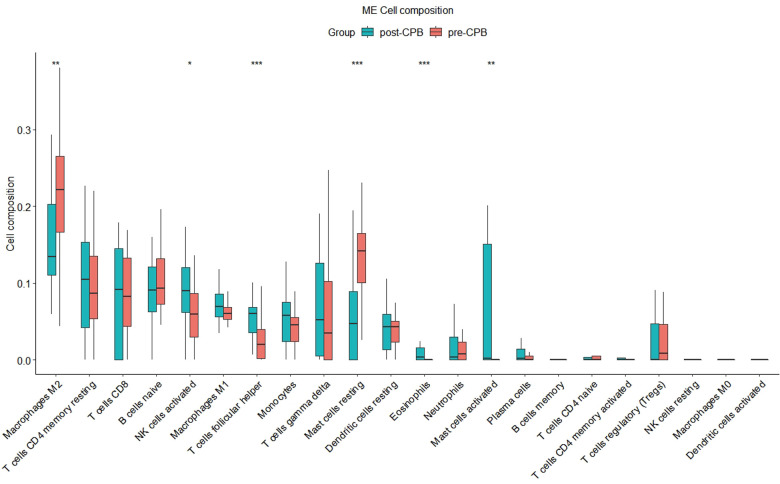
Immune infiltration analysis plot. There was a significant change in macrophages M2 (*P* < 0.01), mast cells resting (*P* < 0.001), NK cells activated (*P* < 0.05), T cells follicular helper (*P* < 0.001), mast cells activated (*P* < 0.01) and eosinophils (*P* < 0.001) in the post-CPB group compared to the pre-CPB group. “*”, “**”, “***” represent *P* < 0.05, *P* < 0.01, *P* < 0.001, respectively.

By using the CIBERSORT R code, we found a significant change in macrophages M2 (*P* < 0.01), mast cells resting (*P* < 0.001), NK cells activated (*P* < 0.05), T cells follicular helper (*P* < 0.001), mast cells activated (*P* < 0.01) and eosinophils (*P* < 0.001) in the post-CPB group compared to the pre-CPB group (Figure 10). Among them, the former two were significantly lower and the last four were significantly higher. By using the CIBERSORTx online analysis website, we found that *CDKN1A* expression was elevated in eosinophils, neutrophils, memory CD4 T cells and activated mast cells (Supplementary file: CIBERSORTx_Job4_output/CIBERSORTxGEP_Job4_GEPs_Filtered.txt).

### Hub gene validation

**Figure 11:**
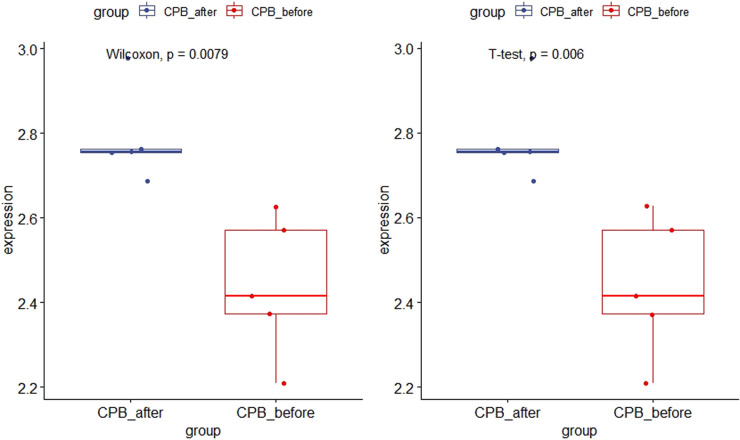
Box plots of CDKN1A gene expression in the validation set. The expression of CDKN1A in the GSE12486 validation dataset was significantly lower in the pre-CPB group than in the post-CPB group (Wilcoxon, *P* = 0.0079; T-test, *P* = 0.006).

**Figure 12:**
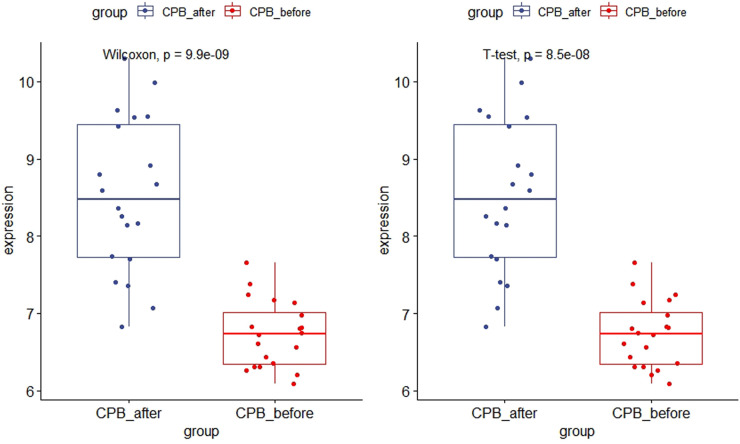
Box plots of CDKN1A gene expression in the GSE 132176 dataset. The expression of CDKN1A was significantly lower in the pre-CPB group than in the post-CPB group (Wilcoxon, *P* = 9.9 × 10^−9^; T-test, *P* = 8.5 × 10^−9^).

**Figure 13:**
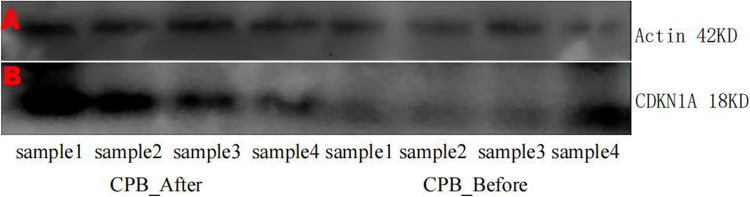
SDS-PAG electrophoregram. (**A**) β-actin (**B**) CDKN1A Line 1–4 are CPB-after samples, Line 5–8 CPB-before samples. Pictures of gels were cropped and juxtaposed and the full length gel pictures could be found in Supplementary Figures 1, 2.

As shown in Figure 11, the expression of *CDKN1A* in the GSE12486 validation dataset was significantly lower in the pre-CPB group than in the post-CPB group(Wilcoxon, *P* = 0.0079; T-test, *P* = 0.006). The same results are shown in the GSE 132176 dataset (Wilcoxon, *P* = 9.9 × 10^−9^; T-test, *P* = 8.5 × 10^−9^) (Figure 12). Western blot analysis also showed that the expression of CDKN1A protein was significantly higher post-CPB than pre-CPB (Figure 13).

## Discussion

In the present study, we performed co-expression network analysis on the GSE132176 dataset, which contains 10 samples of TOF before and after CPB as well as 10 samples of ASD before and after CPB. After identifying the module related to post-CPB, the hub genes in the modules were screened using two methods. *CDKN1A* was obtained as the key gene for inflammation post-CPB in CHD and was verified with the GSE12486 dataset. Disease enrichment analysis indicated that the genes in the brown module were enriched for inflammatory diseases. In addition, *CDKN1A* expression was elevated eosinophils, neutrophils, memory CD4 T cells and activated mast cells. Western blot analysis suggested that CDKN1A expression was significantly higher post-CPB than pre-CPB. Together, these results suggested that *CDKN1A* may be a potential gene that causes complications post-CPB for CHD. Enrichment analysis of functions and signaling pathways by GO and KEGG analyses showed that the genes within the brown module were significantly enriched in inflammation-related genes. Among the top 20 GO terms with *CKNA1A* involvement, the most interesting one was response to corticosteroid (GO:0031960), which belongs to cellular response to lipid (GO:0071396), because corticosteroids are involved in a wide range of physiological systems, such as stress response, immune response regulation, inflammation regulation, carbohydrate metabolism, protein catalysis, blood electrolyte levels and behavior.

CHD is the most common congenital cardiovascular abnormality (33), accounting for 1% of human congenital malformations (34). Cardiac surgery significantly increases the survival rate of patients with CHD (35). CPB is an important aspect of many cardiothoracic surgeries, but it is related to excessive systemic inflammatory response and the release of cardiac biomarkers (36). The mechanism of complications after cardiac surgery CPB is complicated, and there may be many genes involved in its regulation. However, specific biomarkers for accurate diagnostic testing and potential therapeutic targets for personalized treatment have not been fully identified in inflammation following CPB for CHD.

Cyclin dependent kinase (CDK) inhibitors regulate inflammatory cell differentiation and function as well as inflammatory signaling pathways and apoptosis (37). *CDKN1A* is an important member of the CDK inhibitor family, and it belongs to the Cip/Kip family of CDK inhibitors, thereby signifying its name, p21^WAF1/CIP1^ (38). Studies have shown that *CDKN1A* is involved in the inflammatory process in various diseases, but its role varies. *CDKN1A* is essential in lymphotoxin-driven pancreatic inflammation of the pancreas in response to pancreatic injury, and it is involved in the pre-acinar secretion of inflammatory mediators that recruit innate immune cells (39). In sarcoid granulomas, high expression of *CDKN1A* reduces apoptotic events and leads to persistent inflammation (40). Disruption of *CDKN1A* alleviates lung inflammation induced by cigarette smoke, lipopolysaccharide (LPS) and N-formyl-methionyl-leucyl-phenylalanine (fMLP) in mice (41). Although the etiology of idiopathic pulmonary fibrosis (IPF) is uncertain, it is presumed that IPF begins with an initial inflammatory lesion confined to the alveoli and progresses to the chronic inflammation of alveolitis. Kuwano et al. found that *p53* and *CDKN1A* are expressed in proliferating bronchial and alveolar epithelial cells in the lung tissue of all IPF patients (42). Together, these studies suggest that *CDKN1A* plays an active role in chronic inflammation and the incessant state of inflammation. Contrary to the results of the aforementioned studies, Sarfraz et al. found that *CDKN1A*-positive hepatocyte expression correlates with fibrosis staging progression but not with inflammatory grade (43). It has also been shown that *CDKN1A* inhibits rheumatoid inflammation by downregulating the expression of the type I IL-1 receptor (IL-1RI) and inhibiting JNK activity (44). Thus, *CDKN1A* plays different roles in different inflammatory diseases with some positive and negative roles.

*CDKN1A* is also involved in cardiac development. In a mouse model, *Wdfy3* deficiency induces *CDKN1A* promoter activity, which leads to embryonic heart development and consequently to CHD (45). *TBX3* deficiency accelerates apoptosis by directly regulating *CDKN1A* expression in senescent cardiomyocytes (46). Moreover, a hyperglycemic status can lead to reduced proliferation of cardiomyocytes and endocardial cells through overexpression of *CDKN1A* (47). These findings suggest that *CDKN1A* is involved in the formation of cardiac malformations.

Admittedly, there are limitations to this study, as the lack of clinical data in the training dataset prevents us from validating the diagnostic efficiency of *CDKN1A*, coupled with the small number of clinical specimens currently collected and the inability to link closely to the clinic. Therefore, a large number of samples with clinical information need to be collected for more in-depth studies in the future.

## Conclusion

In summary, the present study used a series of bioinformatics analysis methods and Western blot analysis to determine and verify the relationship between *CDKN1A* and CPB for CHD. The present study indicated that *CDKN1A* may be involved in inflammation of post-CPB for CHD. These results have important clinical significance and contribute to the accurate treatment of post-CPB inflammation in patients with CHD.

## Data Availability

The original contributions presented in the study are included in the article/Suplementary Material, further inquiries can be directed to the corresponding author/s.

## References

[1] Guo L Zhao D Zhang R Li S Liu R Wang H A matched case-control study on the association between colds, depressive symptoms during pregnancy and congenital heart disease in northwestern China. Sci Rep. (2019) 9(1):589. 10.1038/s41598-018-36968-y 30679633 PMC6345882

[2] Brueckner M. Heterotaxia, congenital heart disease, and primary ciliary dyskinesia. Circulation. (2007) 115(22):2793–5. 10.1161/CIRCULATIONAHA.107.699256 17548739

[3] Davidson JA Khailova L Treece A Robison J Soranno DE Jaggers J Alkaline phosphatase treatment of acute kidney injury in an infant piglet model of cardiopulmonary bypass with deep hypothermic circulatory arrest. Sci Rep. (2019) 9(1):14175. 10.1038/s41598-019-50481-w 31578351 PMC6775126

[4] Madershahian N Scherner M Pfister R Rudolph T Deppe AC Slottosch I Prophylactic intraoperative tranexamic acid administration and postoperative blood loss after transapical aortic valve implantation. J Cardiothorac Surg. (2015) 10:45. 10.1186/s13019-015-0246-5 25888231 PMC4393600

[5] Zhang Z Wu Y Zhao Y Xiao X Liu J Zhou X. Dynamic changes in HMGB1 levels correlate with inflammatory responses during cardiopulmonary bypass. Exp Ther Med. (2013) 5(5):1523–7. 10.3892/etm.2013.1026 23737912 PMC3671828

[6] Tu Z Tan X Li S Cui J Tu S Jiang L. The therapeutic effect of controlled reoxygenation on chronic hypoxia-associated brain injury. Biol Open. (2019) 8(12):bio039370. 10.1242/bio.039370 31719034 PMC6918765

[7] Lombard FW Mathew JP. Neurocognitive dysfunction following cardiac surgery. Semin Cardiothorac Vasc Anesth. (2010) 14(2):102–10. 10.1177/1089253210371519 20478950

[8] Milovanovic V Bisenic D Mimic B Ali B Cantinotti M Soldatovic I Reevaluating the importance of modified ultrafiltration in contemporary pediatric cardiac surgery. J Clin Med. (2018) 7(12):498. 10.3390/jcm7120498 PMC6306792 30513728

[9] Shen C Gu T Gu L Xiu Z Zhang Z Shi E Change in the perioperative blood glucose and blood lactate levels of non-diabetic patients undergoing coronary bypass surgery. Exp Ther Med. (2013) 6(5):1220–4. 10.3892/etm.2013.1268 24223647 PMC3820790

[10] Roth-Isigkeit A Borstel TV Seyfarth M Schmucker P. Perioperative serum levels of tumour-necrosis-factor alpha (TNF-alpha), IL-1 beta, IL-6, IL-10 and soluble IL-2 receptor in patients undergoing cardiac surgery with cardiopulmonary bypass without and with correction for haemodilution. Clin Exp Immunol. (1999) 118(2):242–6. 10.1046/j.1365-2249.1999.01050.x 10540185 PMC1905422

[11] Kirklin JK Westaby S Blackstone EH Kirklin JW Chenoweth DE Pacifico AD. Complement and the damaging effects of cardiopulmonary bypass. J Thorac Cardiovasc Surg. (1983) 86(6):845–57. 10.1016/S0022-5223(19)39061-0 6606084

[12] Herbst-Rodrigues MV Carvalho VO Abrahao LH Nozawa E Feltrim MI Gomes-Galas FR. Alveolar recruitment maneuver in refractory hypoxemia and lobar atelectasis after cardiac surgery: a case report. J Cardiothorac Surg. (2012) 7:58. 10.1186/1749-8090-7-58 22726992 PMC3567961

[13] Rumsfeld JS MaWhinney S McCarthy M Shroyer AL VillaNueva CB O'Brien M Health-related quality of life as a predictor of mortality following coronary artery bypass graft surgery. Participants of the Department of Veterans Affairs Cooperative Study Group on Processes, Structures, and Outcomes of Care in Cardiac Surgery. JAMA. (1999) 281(14):1298–303. 10.1001/jama.281.14.1298 10208145

[14] Qu C Wang XW Huang C Qiu F Xiang XY Lu ZQ. High mobility group box 1 gene polymorphism is associated with the risk of postoperative atrial fibrillation after coronary artery bypass surgery. J Cardiothorac Surg. (2015) 10:88. 10.1186/s13019-015-0301-2 26109393 PMC4480995

[15] Ivancan V Colak Z Konosic S Gabelica R Anic D Belina D Effects of corticosteroids after total reapair of congenital heart desease with extracorporeal circulation. J Cardiothorac Surg. (2013) 8(Suppl 1):O6. 10.1186/1749-8090-8-S1-O6

[16] Kirklin JK. Prospects for understanding and eliminating the deleterious effects of cardiopulmonary bypass. Ann Thorac Surg. (1991) 51(4):529–31. 10.1016/0003-4975(91)90302-7 2012410

[17] Schultz JM Karamlou T Swanson J Shen I Ungerleider RM. Hypothermic low-flow cardiopulmonary bypass impairs pulmonary and right ventricular function more than circulatory arrest. Ann Thorac Surg. (2006) 81(2):474–80; discussion 480. 10.1016/j.athoracsur.2005.06.041 16427835

[18] Rungatscher A Tessari M Stranieri C Solani E Linardi D Milani E Oxygenator is the main responsible for leukocyte activation in experimental model of extracorporeal circulation: a cautionary tale. Mediators Inflamm. (2015) 2015:484979. 10.1155/2015/484979 26063972 PMC4434202

[19] Larson DF Horak K. Macrophage migration inhibitory factor: controller of systemic inflammation. Crit Care. (2006) 10(2):138. 10.1186/cc4899 16677407 PMC1550887

[20] MacCallum NS Gordon SE Quinlan GJ Evans TW Finney SJ. Systemic inflammatory response syndrome post cardiac surgery: a useful concept? Critical Care. (2008) 12(Suppl 2):P258. 10.1186/cc6479

[21] Nazarieh M Helms V. Topcontrol: a tool to prioritize candidate disease-associated genes based on topological network features. Sci Rep. (2019) 9(1):19472. 10.1038/s41598-019-55954-6 31857653 PMC6923402

[22] Pavlidis P Noble WS. Analysis of strain and regional variation in gene expression in mouse brain. Genome Biol. (2001) 2(10):RESEARCH0042. 10.1186/gb-2001-2-10-research0042 11597334 PMC57797

[23] Siska C Bowler R Kechris K. The discordant method: a novel approach for differential correlation. Bioinformatics (Oxford, England). (2016) 32(5):690–6. 10.1093/bioinformatics/btv633 26520855 PMC5006287

[24] Xiao KW Li JL Zeng ZH Liu ZB Hou ZQ Yan X Monocytes affect bone mineral density in pre- and postmenopausal women through ribonucleoprotein complex biogenesis by integrative bioinformatics analysis. Sci Rep. (2019) 9(1):17290. 10.1038/s41598-019-53843-6 31754224 PMC6872746

[25] Langfelder P Horvath S. WGCNA: an R package for weighted correlation network analysis. BMC Bioinformatics. (2008) 9:559. 10.1186/1471-2105-9-559 19114008 PMC2631488

[26] Holtman IR Raj DD Miller JA Schaafsma W Yin Z Brouwer N Induction of a common microglia gene expression signature by aging and neurodegenerative conditions: a co-expression meta-analysis. Acta Neuropathol Commun. (2015) 3:31. 10.1186/s40478-015-0203-5 26001565 PMC4489356

[27] Raggi F Cangelosi D Becherini P Blengio F Morini M Acquaviva M Transcriptome analysis defines myocardium gene signatures in children with ToF and ASD and reveals disease-specific molecular reprogramming in response to surgery with cardiopulmonary bypass. J Transl Med. (2020) 18(1):21. 10.1186/s12967-020-02210-5 31924244 PMC6954611

[28] Ghorbel MT Cherif M Mokhtari A Bruno VD Caputo M Angelini GD. Off-pump coronary artery bypass surgery is associated with fewer gene expression changes in the human myocardium in comparison with on-pump surgery. Physiol Genomics. (2010) 42(1):67–75. 10.1152/physiolgenomics.00174.2009 20332183 PMC2888559

[29] Langfelder P Horvath S. Fast R functions for robust correlations and hierarchical clustering. J Stat Softw. (2012) 46(11):i11. 10.18637/jss.v046.i11 23050260 PMC3465711

[30] Szklarczyk D Gable AL Lyon D Junge A Wyder S Huerta-Cepas J STRING V11: protein-protein association networks with increased coverage, supporting functional discovery in genome-wide experimental datasets. Nucleic Acids Res. (2019) 47(D1):D607–13. 10.1093/nar/gky1131 30476243 PMC6323986

[31] Shannon P Markiel A Ozier O Baliga NS Wang JT Ramage D Cytoscape: a software environment for integrated models of biomolecular interaction networks. Genome Res. (2003) 13(11):2498–504. 10.1101/gr.1239303 14597658 PMC403769

[32] Zhou Y Zhou B Pache L Chang M Khodabakhshi AH Tanaseichuk O Metascape provides a biologist-oriented resource for the analysis of systems-level datasets. Nat Commun. (2019) 10(1):1523. 10.1038/s41467-019-09234-6 30944313 PMC6447622

[33] Li X Qiu J Pan M Zheng D Su Y Wei M Correlation between congenital heart disease complicated with pulmonary artery hypertension and circulating endothelial cells as well as endothelin-1. Int J Clin Exp Pathol. (2015) 8(9):10743–51. PMID: ; PMCID: 26617785 PMC4637600

[34] Xiao F Zhuang J Zhou CB Chen JM Cen JZ Xu G Assessing the impact of total extracorporeal circulation on hemodynamics in an ovine fetal model. Exp Ther Med. (2017) 14(3):2709–15. 10.3892/etm.2017.4831 28962216 PMC5609285

[35] Yin CH Yan J Li SJ Li DY Wang Q Wang ES. Effect analysis of repeat sternotomy in pediatric cardiac operations. J Cardiothorac Surg. (2015) 10:179. 10.1186/s13019-015-0381-z 26621353 PMC4666069

[36] Kahraman A Mutlu E Aldağ M. ADMA, SDMA and L-arginine may be novel targets in pharmacotherapy for complications due to cardiopulmonary bypass. J Med Biochem. (2017) 36(1):8–17. 10.1515/jomb-2016-0025 28680344 PMC5471654

[37] Leitch AE Haslett C Rossi AG. Cyclin-dependent kinase inhibitor drugs as potential novel anti-inflammatory and pro-resolution agents. Br J Pharmacol. (2009) 158:1004–16. 10.1111/j.1476-5381.2009.00402.x 19775281 PMC2785523

[38] Deng T Yan G Song X Xie L Zhou Y Li J Deubiquitylation and stabilization of p21 by USP11 is critical for cell-cycle progression and DNA damage responses. Proc Natl Acad Sci USA. (2018) 115(18):4678–83. 10.1073/pnas.1714938115 29666278 PMC5939064

[39] Seleznik GM Reding T Peter L Gupta A Steiner SG Sonda S Development of autoimmune pancreatitis is independent of CDKN1A/p21-mediated pancreatic inflammation. Gut. (2018) 67(9):1663–73. 10.1136/gutjnl-2016-313458 28774888

[40] Xaus J Besalduch N Comalada M Marcoval J Pujol R Mañá J. High expression of p21 Waf1 in sarcoid granulomas: a putative role for long-lasting inflammation. J Leukoc Biol. (2003) 74(2):295–301. 10.1189/jlb.1202628 12885947

[41] Yao H Yang SR Edirisinghe I Rajendrasozhan S Caito S Adenuga D Disruption of p21 attenuates lung inflammation induced by cigarette smoke, LPS, and fMLP in mice. Am J Respir Cell Mol Biol. (2008) 39(1):7–18. 10.1165/rcmb.2007-0342OC 18239191 PMC2440259

[42] Kuwano K Kunitake R Kawasaki M Nomoto Y Hagimoto N Nakanishi Y P21Waf1/Cip1/Sdi1 and p53 expression in association with DNA strand breaks in idiopathic pulmonary fibrosis. Am J Respir Crit Care Med. (1996) 154(2 Pt 1):477–83. 10.1164/ajrccm.154.2.8756825 8756825

[43] Sarfraz S Hamid S Siddiqui A Hussain S Pervez S Alexander G. Altered expression of cell cycle and apoptotic proteins in chronic hepatitis C virus infection. BMC Microbiol. (2008) 8:133. 10.1186/1471-2180-8-133 18680610 PMC2518161

[44] Nonomura Y Nagasaka K Hagiyama H Sekine C Nanki T Tamamori-Adachi M Direct modulation of rheumatoid inflammatory mediator expression in retinoblastoma protein-dependent and -independent pathways by cyclin-dependent kinase 4/6. Arthritis Rheum. (2006) 54(7):2074–83. 10.1002/art.21927 16802342

[45] Zhang S Song Z An L Liu X Hu XW Naz A WD40 Repeat and FYVE domain containing 3 is essential for cardiac development. Cardiovasc Res. (2019) 115(8):1320–31. 10.1093/cvr/cvy285 30428088

[46] Cao M Zhu B Sun Y Zhao X Qiu G Fu W TBX3 Deficiency accelerates apoptosis in cardiomyoblasts through regulation of P21 expression. Life Sci. (2019) 239:117040. 10.1016/j.lfs.2019.117040 31704448

[47] Scott-Drechsel DE Rugonyi S Marks DL Thornburg KL Hinds MT. Hyperglycemia slows embryonic growth and suppresses cell cycle via cyclin D1 and p21. Diabetes. (2013) 62(1):234–42. 10.2337/db12-0161 23193186 PMC3526024

